# Unimodal Versus Bimodal EEG-fMRI Neurofeedback of a Motor Imagery Task

**DOI:** 10.3389/fnhum.2017.00193

**Published:** 2017-04-20

**Authors:** Lorraine Perronnet, Anatole Lécuyer, Marsel Mano, Elise Bannier, Fabien Lotte, Maureen Clerc, Christian Barillot

**Affiliations:** ^1^INRIA, VisAGeS Project TeamRennes, France; ^2^Centre National de la Recherche Scientifique, IRISA, UMR 6074Rennes, France; ^3^Institut National de la Santé et de la Recherche Médicale, U1228Rennes, France; ^4^Université Rennes 1Rennes, France; ^5^INRIA, Hybrid Project TeamRennes, France; ^6^CHU RennesRennes, France; ^7^Inria, Potioc Project TeamTalence, France; ^8^LaBRIBordeaux, France; ^9^Inria, Athena Project TeamSophia Antipolis, France; ^10^Université Côte d'AzurNice, France

**Keywords:** neurofeedback, EEG, fMRI, motor imagery, multimodal

## Abstract

Neurofeedback is a promising tool for brain rehabilitation and peak performance training. Neurofeedback approaches usually rely on a single brain imaging modality such as EEG or fMRI. Combining these modalities for neurofeedback training could allow to provide richer information to the subject and could thus enable him/her to achieve faster and more specific self-regulation. Yet unimodal and multimodal neurofeedback have never been compared before. In the present work, we introduce a simultaneous EEG-fMRI experimental protocol in which participants performed a motor-imagery task in unimodal and bimodal NF conditions. With this protocol we were able to compare for the first time the effects of unimodal EEG-neurofeedback and fMRI-neurofeedback versus bimodal EEG-fMRI-neurofeedback by looking both at EEG and fMRI activations. We also propose a new feedback metaphor for bimodal EEG-fMRI-neurofeedback that integrates both EEG and fMRI signal in a single bi-dimensional feedback (a ball moving in 2D). Such a feedback is intended to relieve the cognitive load of the subject by presenting the bimodal neurofeedback task as a single regulation task instead of two. Additionally, this integrated feedback metaphor gives flexibility on defining a bimodal neurofeedback target. Participants were able to regulate activity in their motor regions in all NF conditions. Moreover, motor activations as revealed by offline fMRI analysis were stronger during EEG-fMRI-neurofeedback than during EEG-neurofeedback. This result suggests that EEG-fMRI-neurofeedback could be more specific or more engaging than EEG-neurofeedback. Our results also suggest that during EEG-fMRI-neurofeedback, participants tended to regulate more the modality that was harder to control. Taken together our results shed first light on the specific mechanisms of bimodal EEG-fMRI-neurofeedback and on its added-value as compared to unimodal EEG-neurofeedback and fMRI-neurofeedback.

## Introduction

Neurofeedback (NF) is a technique that consists in feeding back information to an individual about his/her brain activity in real-time in order for him/her to learn to better control an aspect of it (Hammond, 2011; Birbaumer et al., 2013). Specific changes in emotional, cognitive, or behavioral functions are expected to occur along with NF training, which makes NF a promising tool for brain rehabilitation of patients with neurological or psychiatric disorders (Birbaumer et al., 2009; Sulzer et al., 2013; Linden, 2014; Linden and Turner, 2016) and for peak performance training of healthy subjects (Vernon, 2005; Gruzelier, 2014a,b; Scharnowski and Weiskopf, 2015). From the advent of NF in the early sixties up to now, most NF studies have relied on the use of EEG for measuring the brain activity. While EEG is inexpensive and benefits from a high temporal resolution (order of the millisecond), it is particularly sensitive to noise and lacks specificity because of its low spatial resolution (order of the centimeter) and the fact that source localization from EEG suffers from an ill-posed inverse problem (Baillet et al., 2001; Grech et al., 2008). Combining EEG with other modalities could allow to extract richer information about the ongoing brain activity and therefore enable to develop more efficient NF protocols. In the recent years, fMRI, functional near-infrared spectroscopy (fNIRS), and magnetoencephalography (MEG) have started to be exploited for the purpose of NF (Sudre et al., 2011; Masahito Mihara et al., 2012; Sulzer et al., 2013; Thibault et al., 2016). Most noteworthy, the field of fMRI-NF has grown exponentially during the last 10 years (Sulzer et al., 2013) and contributed in reviving NF research. However, if fMRI-NF gives access to the self-regulation of deep brain regions at high spatial resolution (order or the millimeter), it is at the price of a low temporal resolution (order of the second). In the context of NF, combining EEG and fMRI enables to return to a subject two signals at the same time, one that contains temporally fine information about brain oscillations and one that contains spatially fine information about specific brain regions. The simultaneous combination of EEG-NF and fMRI-NF was introduced for the first time by Zotev et al. (2014) who made a proof-of-concept application of this new type of NF in the training of emotional self-regulation. In their pioneering work, the authors hypothesized that bimodal EEG-fMRI-NF could be more efficient than EEG-NF or fMRI-NF performed alone. However, to our knowledge, this hypothesis has not been studied so far.

As NF, brain computer interfaces (BCI) also rely on a closed-loop that exploits brain activity in real-time. Traditional BCIs, so called assistive BCIs, are intended for the purpose of control and communication of an external object, while NF is intended to allow an individual to acquire control over him/herself (Wyckoff and Birbaumer, 2014; Perronnet et al., 2016). This distinction has tended to fade since the development of restorative/rehabilitative BCIs which target brain rehabilitation applications like NF (Soekadar et al., 2011; Chaudhary et al., 2016; Perronnet et al., 2016). In the BCI community, the field of hybrid BCI has recently emerged (Pfurtscheller et al., 2010; Amiri et al., 2013), the term “hybrid” referring to a multimodal combination of sensors. A hybrid BCI is defined as the combination of two BCIs or of at least a BCI and another system such as another biofeedback system (like an electromyogram for example; Pfurtscheller et al., 2010). They can be designed to work simultaneously or sequentially. Their purpose is mostly to augment the usability and/or the performance of the BCI. Most of the hybrid BCIs combining two BCIs that have been proposed in the literature relied only on EEG paradigms, but some hybrid BCI combining EEG and fNIRS have also been proposed (Fazli et al., 2012; Buccino et al., 2016) and have shown enhanced performance. These encouraging results suggest that using hybrid approaches for NF could enhance the efficiency of unimodal approaches.

Protocols targeting motor imagery (MI) patterns are one of the most studied applications of NF and BCI research because of their potential for stroke rehabilitation (Sharma et al., 2006). To this day, such NF protocols have been explored with all existing NF modalities: EEG (Birbaumer et al., 2009; Shindo et al., 2011; Silvoni et al., 2011; Ramos-Murguialday et al., 2013; Pichiorri et al., 2015), fMRI (Sitaram et al., 2012; Liew et al., 2015; Linden and Turner, 2016), and even the most recent ones fNIRS (Mihara et al., 2013; Kober et al., 2014) and MEG (Foldes et al., 2015). A recent study by Zich et al. (2015) showed that MI-based EEG-NF allowed subjects not only to generate stronger EEG response at the motor electrodes, but also that the BOLD activity observed in the sensorimotor regions in simultaneous fMRI recordings was higher during MI with EEG-NF as compared with MI training alone. Interestingly enough, the study revealed that the contralateral activity in EEG and fMRI were correlated while the laterality patterns were not. The finding that EEG and fMRI signatures of MI are not redundant suggests a potential for bimodal EEG-fMRI-NF. To our knowledge no bimodal MI-based EEG-fMRI-NF protocol has already been proposed. The authors (Zich et al., 2015) particularly stressed the need of conducting an exhaustive comparison of unimodal and bimodal neurofeedback in order to understand the specific contribution of each modality: “*only a systematic within-subject comparison using simultaneous EEG-fMRI data acquisition and providing fMRI-based feedback, EEG-based feedback and a hybrid feedback based on both modalities, would provide exact information about the validity of each recording modality.”*

In the present work, we introduce an MI-based EEG-fMRI-NF protocol and compare its effects with EEG-NF and fMRI-NF by looking at the MI-related EEG and fMRI activity patterns elicited in each NF condition. Additionally, we introduce a hybrid EEG-fMRI-NF metaphor that integrates both EEG and fMRI signals simultaneously in a single bi-dimensional feedback. We assume that in this way, subjects are more likely to perceive the NF task as one regulation task instead of two and that this might relieve their cognitive load (Gaume et al., 2016).

## Methods

### Experimental procedure

The study was conducted at the Neurinfo platform (CHU Pontchaillou, Rennes France) and was approved by the Institutional Review Board. Ten right-handed NF-naïve healthy volunteers with no prior MI-NF experience (mean age: 28 ± 5.7 years, two females) participated in the study. Throughout the whole experiment, the participants were lying down in the MR bore and wearing a 64 channel MR-compatible EEG cap.

#### Instructions

**Figure 1:**
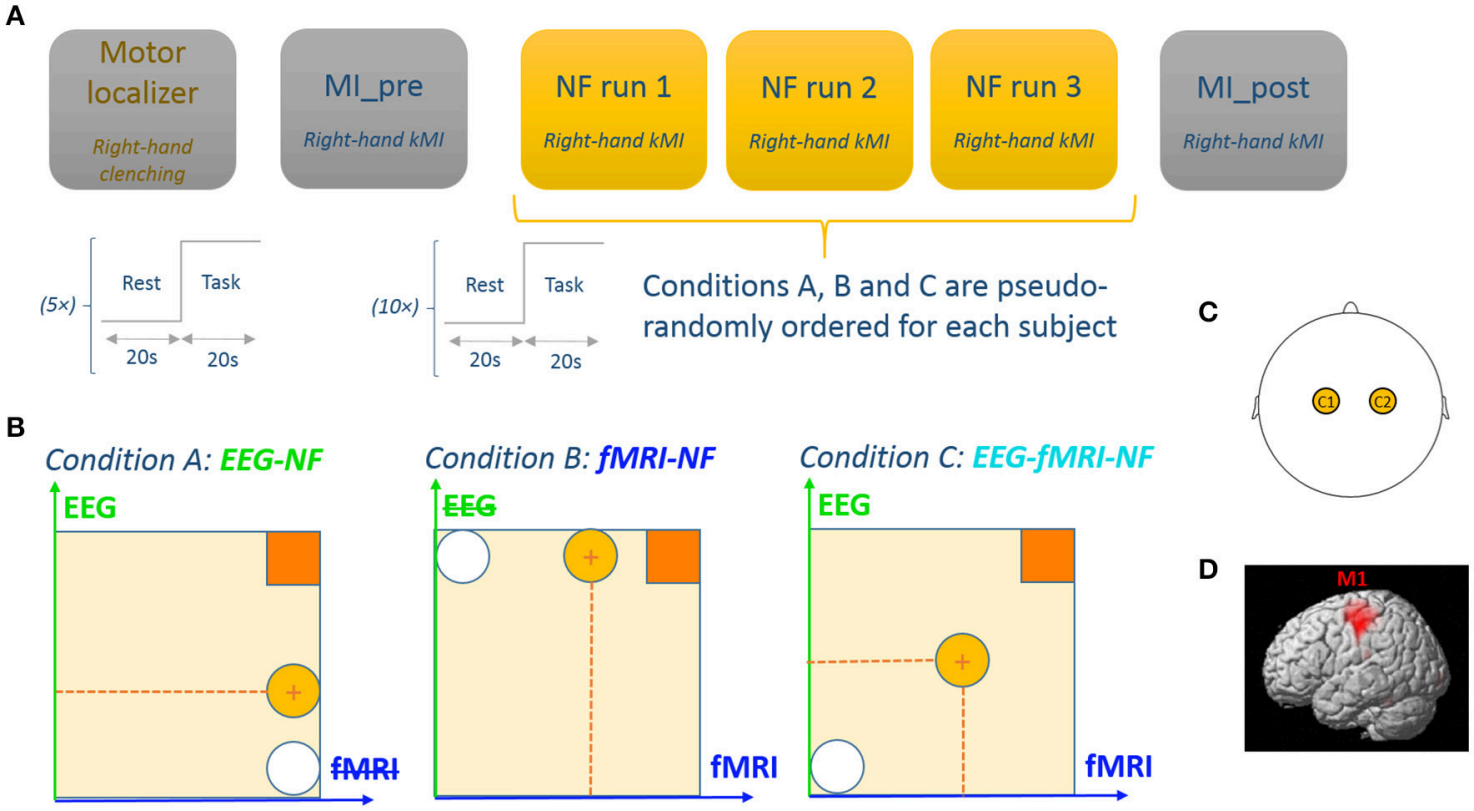
**Experimental procedure. (A)** The experimental protocol consisted of 6 EEG-fMRI runs: a motor localizer run, a motor imagery run without NF, three NF runs with different NF conditions, and a post motor imagery run without NF. Each run consisted of a block design with 20 s blocks. **(B)** Feedback display for each experimental conditions. Feedback was represented by a ball moving in 1 dimension (condition A and B) or 2 dimensions (condition C). The white circle represents the starting ball position and the yellow circle depicts a possible ball position. Participants are instructed to get the ball closer to the square in the upper-right by performing kinesthetic motor imagery. **(C)** For the EEG feature, we used a laterality index between C1 and C2. **(D)** For the fMRI feature we used a laterality index between left M1 and right M1.

After signing an informed consent form describing the MR environment, the participants were verbally informed about the goal of the study and of the protocol. They were instructed that during the NF runs, they would be presented with a ball moving in one or two dimensions according to the activity in their motor regions measured with EEG and/or fMRI. They were told that they would have to bring the ball closer to the square in the top-right corner (see Figure 1) by imagining that they were moving their right-hand. This instruction was reminded in written form on the screen at the beginning of each NF run. More specifically we explained that they would need to perform kinesthetic motor imagery (kMI) of their right-hand in order to control the ball. Kinesthetic motor imagery was defined as trying to feel the sensation of the motion rather than visualizing it. We suggested different MI strategies to the participants such as imagining hand clenching or finger tapping, imagining that they were playing the piano, or imagining a hand motion that they were used to perform. They were encouraged to try several strategies and stick with the one that worked best. More specifically, they were informed that the EEG and fMRI measures that would be used to display the feedback were laterality indices. This implied that they would have to maximize the activity in their right-hand region while minimizing it in the left-hand region in order to reach the NF target (get the ball closer to the upper-right square), so that bimanual imagination would not enable them to control the feedback. Participants were informed about the nature of EEG and fMRI signal, and specifically about the 4–6 s delay of the hemodynamic response. These general instructions were given verbally at the beginning of the experiment and reminded later if the participant asked for it. Before each NF run, the participants received verbal notice about which dimension/s (horizontal and/or vertical) was/were going to be active in the upcoming run. Participants were asked not to move at all, especially during the course of a run. Video monitoring of the inside of the MR tube allowed to check for whole-body movements of the participant.

#### Protocol

The experimental protocol consisted of six EEG-fMRI runs employing a block-design alternating 20 s of rest and 20 s of task (see Figure 1):

- A motor localizer run (*MLOC*) lasting 5 min 20 s
- A preliminary motor-imagery run without NF (*MI_pre*) lasting 3 min 20 s
- Three NF runs (*NF1, NF2, NF3*) lasting 6 min 40 s each and corresponding to three different feedback modality conditions (A: *EEG-NF*; B: *fMRI-NF*; C: *EEG-fMRI-NF*) whose order was counter-balanced across participants
- And a final motor-imagery run without NF (*MI_post*) lasting 3 min 20 s.

During rest, the screen displayed a white cross and participants were asked to concentrate on the cross and not on the passed or upcoming task. During task, the screen displayed a cue (“move right”/“imagine right”) as well as the NF ball and target during NF runs. At the end of the experiment, the participants were asked to fill out a questionnaire about their performance, motivation, fatigue, interest, difficulty in performing the NF tasks, and specific questions about the bimodal *EEG-fMRI-NF* run. For two participants out of the 10, *MI_pre* and *MI_post* could not be acquired due to technical reasons.

During the active blocks of the motor localizer run the participants were asked to perform right-hand clenching at 1 Hz. Immediately at the end of this run, the corresponding activation map computed by the MR vendor console (eva_series GLM file) was used to define a ROI mask over the left primary motor cortex (M1) as a 9 × 9 × 3 voxel (18 × 18 × 12 mm^3^) cube centered on the left M1 voxel with the maximum *t*-value. The right M1 ROI was defined by taking the left M1 ROI symmetric in the sagittal plane. These ROIs were used later during the NF runs for computing the fMRI NF feature.

During the active blocks of the *MI_pre* run, participants were asked to perform kMI of their right-hand. They were suggested to imagine their right-hand clenching by trying to recall the sensation they had in their right hand when actually executing the movement in the previous run. The goal of this run was for the participants to practice motor imagery. The data from this run was also intended to be used later for assessing the NF learning effect if any.

During the active blocks of the NF runs (*NF1, NF2, NF3*), the screen displayed a white ball moving in the vertical (condition A), or horizontal (condition B) or both dimensions (condition C) and a square in the top-right corner of the screen representing the target to reach. The same feedback metaphor was used during unimodal and bimodal feedback in order to prevent the occurrence of a confounding effect from the feedback metaphor. The participants were instructed to bring the ball closer to the square by performing kMI of their right hand. The ball abscissa depicted a BOLD laterality index (signal difference) between the left and right M1 ROI (Chiew et al., 2012) and was updated every repetition time (TR = 2 s). In a similar fashion, the ball ordinate depicted the laterality index (see Section Real-Time Data Processing) between electrodes C1 and C2 in the μ (8–12 Hz) band and was updated every 250 ms. Figure 1 illustrates the experimental protocol.

Eventually, during the active blocks of the *MI_post* run, participants were asked to perform motor imagery with the strategy that they found out worked best throughout the NF runs. This run was intended to be used as a transfer run which purpose is that the participant learns to self-regulate in absence of any NF. The data was also intended to be used for assessing the NF learning effect between *MI_pre* and *MI_post*.

### Data acquisition/technical setup

EEG and fMRI data were simultaneously acquired with a 64-channel MR-compatible EEG solution from Brain Products (Brain Products GmbH, Gilching, Germany) and a 3T Verio Siemens scanner (VB17) with a 12–channel head coil. Foam pads were used to restrict head motion. EEG data was sampled at 5 kHz with FCz as the reference electrode and AFz as the ground electrode. fMRI acquisitions were performed using echo-planar imaging (EPI) with the following parameters: repetition time (TR)/echo time (TE) = 2000/23 ms, 210 × 210 mm^2^ FOV, voxel size = 2 × 2 × 4 mm^3^, matrix size = 105 × 105, 32 slices, flip angle = 90°). Visual instructions and feedback were transmitted using the NordicNeurolab hardware and presented to the participant via an LCD screen and a rear-facing mirror fixed on the coil.

As a structural reference for the fMRI analysis, a high resolution 3D T1 MPRAGE sequence was acquired with the following parameters: TR/TI/TE = 1900/900/2.26 ms, GRAPPA 2, 256 × 256 mm^2^ FOV and 176 slabs, 1 × 1 × 1 mm^3^ voxel size, flip angle = 9°.

**Figure 2:**
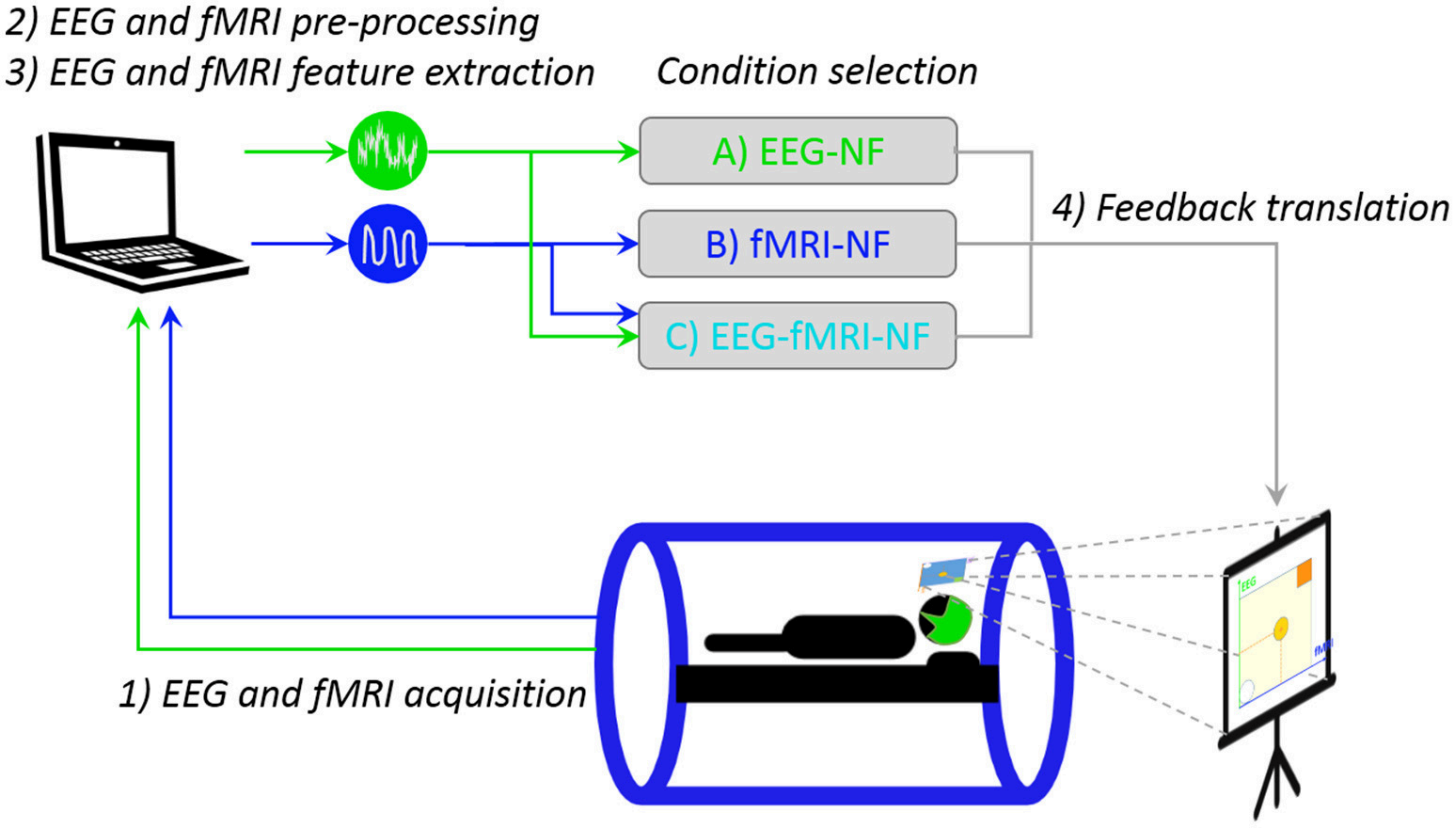
**Real-time multimodal EEG/fMRI-NF setup**. The participant is lying in the MR tube with a 64-channel MR-compatible EEG cap. EEG and fMRI are simultaneously acquired then pre-processed with custom Matlab code. The EEG and fMRI laterality features are computed and eventually translated as a displacement of the ball on the stimulation screen, the image of which is projected on the mirror mounted on the head coil. Icons made by Freepik from www.flaticon.com.

Our multimodal EEG/fMRI-NF system (Mano et al., 2017) integrates EEG and fMRI data streams via a TCP/IP socket. The EEG data is pre-processed with BrainVision Review (Brain Products GmbH, Gilching, Germany) software for gradient and ballistocardiogram (BCG) artifact correction (see Section Real-Time Data Processing) and sent to Matlab (The MathWorks, Inc., Natick, Massachussets, United States) for further processing. The fMRI data is pre-processed online for slice-time correction and motion correction with custom Matlab code adapted from SPM8 (FIL, Wellcome Trust Centre for Neuroimaging, UCL, London, UK). EEG and fMRI NF features are then computed and translated as feedback (vertical and horizontal displacement of the ball) with Psychtoolbox (Kleiner et al., 2007). The fMRI NF dimension is updated every TR (2 s, 0.5 Hz), while the EEG NF dimension is updated at 8 Hz. Figure 2 illustrates the real-time multimodal EEG/fMRI-NF setup.

### Real-time data processing

Online gradient artifact correction and BCG correction of the EEG data were done in BrainVision Recview (Brain Products GmbH, Gilching, Germany) software. The gradient artifact correction in Recview is based on the average artifact subtraction method (Allen et al., 2000). We used an artifact subtraction template of 2,000 ms and four templates for template drift correction. The data was then down-sampled to 200 Hz and low pass filtered at 50 Hz (48 db slope) with a Butterworth filter. The data were subsequently corrected for BCG artifact (Allen et al., 1998). The pulse model was searched in the first 15 s of the data. The pulse detection was based on a moving template matching approach with minimal pulse period of 800 ms, minimum correlation threshold of 0.7, and amplitude ratio range from 0.6 to 1.2 relative to the pulse model. For pulse correction, a moving template was computed by averaging the 10 previously detected pulses, and the correction was done on a window length of [−100 ms, 700 ms] relatively to the R-peak. This corrected data was then sent to Matlab. Every 125 ms the EEG laterality index was computed according to the following equation:

$$\begin{array}{r} eeg_{lat}\left(t\right)=\frac{nLbp\left(t\right)-nRbp\left(t\right)}{nLbp\left(t\right)+nRbp\left(t\right)} \end{array}$$

Where *nLbp*(*t*) [respectively, *nRbp*(*t*)] is the normalized band power in the μ (8–12 Hz) band at the left motor electrode C1 (respectively at the right motor electrode C2) at time *t*. We defined *nLbp* and *nRbp* so that they would be higher than 1 when a desynchronization happened at the corresponding electrode:

$$\begin{array}{r} nLbp\left(t\right)=\bar{Lbp\left(previous\text{\_}rest\right)}/Lbp\left(t\right) \end{array}$$

$$\begin{array}{r} nRbp\left(t\right)=\bar{Rbp\left(previous\text{\_}rest\right)}/Rbp\left(t\right) \end{array}$$

Where *Lbp*(*t*) [respectively, *Rbp*(*t*)] is the band power in the μ band computed at a bipolar derivation around C1 (respectively C2; Neuper et al., 2006) on a 2 s window and $\bar{Lbp\left(previous\text{\_}rest\right)}$ [respectively, $\bar{Rbp\left(previous\text{\_}rest\right)}$] is the left baseline (respectively the right baseline) obtained by averaging the *Lbp* values (respectively the *Rbp* values) over the previous rest block ignoring the first and last second of the block. Eventually, the EEG laterality index *eeg*_*lat*_(*t*) was translated as the ordinate of the ball.

The fMRI signal was pre-processed online for motion correction, slice-time correction and then the fMRI laterality index was computed according to the following definition (Chiew et al., 2012):

$$\begin{array}{r} fMRI_{lat}\left(v\right)=B_{left}\left(v\right)/\bar{B_{left}\left(previous_{rest}\right)}\\ -\;B_{right}\left(v\right)/\bar{B_{right}\left(previous_{rest}\right)} \end{array}$$

$$\begin{array}{l} \frac{B_{left}\left(v\right)}{\bar{B_{left}\left(previous\text{\_}rest\right)}}-\;\frac{B_{right}\left(v\right)}{\bar{B_{right}\left(previous\text{\_}rest\right)}} \end{array}$$

Where *B*_*left*_(*v*) [respectively, *B*_*right*_(*v*)] is the average of the BOLD signal in the left (respectively right) ROI at volume *v*, and $\bar{B_{left}\left(previous\text{\_}rest\right)}$ [respectively, $\bar{B_{right}\left(previous\text{\_}rest\right)}$] is the left baseline obtained by averaging the signal in the left (respectively right) ROI over the last six volumes (to account for hemodynamic delay) of the previous rest block. The fMRI laterality index was then smoothed by averaging it over the last three volumes and translated as the abscissa of the ball.

### Working hypotheses

Our goal is to compare the level of MI-related EEG and fMRI activations elicited during *EEG-NF, fMRI-NF* and *EEG-fMRI-NF*. We have made assumptions that are not specific to motor imagery and EEG and fMRI but can be defined for any brain pattern and pair of brain imaging modalities (*P, Q*). These assumptions concern the order relations of activation levels in a given modality *P* when NF of this modality is given (P-NF), when NF of another modality is given (Q-NF), and when NF of this modality and another is given (P-Q-NF). We hypothesize that:

(H1) *Generalized NF effect: Activation level in a given modality is significant when NF of this modality is displayed, may it be alone or together with another modality (for the specific application to EEG and fMRI, see below the corresponding refined assumptions 1.1, 1.2, 2.1, 2.2)*.
(H2) *Direct NF effect: As a corollary of the generalized NF effect, activation level in a given modality should be higher when NF of this modality is displayed than when it is not displayed, because in the former case the subject has access to it and can thus better and directly regulate it (1.3, 1.4, 2.3, 2.4)*.
(H3) *Compromise effect: Activation level in a given modality is higher or equal when NF of this modality is displayed alone as when it is displayed with another modality, because in the latter case the subject will also try to regulate the other modality (1.5, 2.5)*.

Let *eeg(NF_condition*) be the MI-related EEG activity pattern during *NF_condition* and *fmri(NF_condition)* the MI-related fMRI activity pattern during *NF_condition*. Applying these general assumptions to MI-related EEG and fMRI activations elicited during EEG-NF, fMRI-NF, and EEG-fMRI-NF and breaking them in unitary order relations, these yields the following refined assumptions (the ones underlined correspond to the assumptions that we validated in the present study):

1.1 $\underline{eeg(EEG-NF)>>0}$: *MI-related EEG activations are significant during EEG-NF*
1.2 $\underline{eeg(EEG-fMRI-NF)>>0}$: *MI-related EEG activations are significant during EEG-fMRI-NF*
1.3 eeg(EEG-NF) > eeg(fMRI-NF): *MI-related EEG activations are higher during EEG-NF than during fMRI-NF*
1.4 eeg(EEG-fMRI-NF) > eeg(fMRI-NF): *MI-related EEG activations are higher during EEG-fMRI-NF than during fMRI-NF*
1.5 eeg(EEG-NF) ≥ eeg(EEG-fMRI-NF): *MI-related EEG activations are higher or equal during EEG-NF than during EEG-fMRI-NF*
2.1 $\underline{fmri(fMRI-NF)>>0}$**: MI-related fMRI activations are significant during fMRI-NF**
2.2 $\underline{fmri(EEG-fMRI-NF)>>0}$**: MI-related fMRI activations are significant during EEG-fMRI-NF**
2.3 fmri(fMRI-NF) > fmri(EEG-NF): *MI-related fMRI activations are higher during fMRI-NF than during EEG-NF*
2.4 $\underline{fmri(EEG-fMRI-NF)>fmri(EEG-NF)}$: *MI-related fMRI activations are higher during EEG-fMRI-NF than during EEG-NF*
2.5 fmri(fMRI-NF) ≥ fmri(EEG-fMRI-NF): *MI-related fMRI activations are higher or equal during fMRI-NF than during EEG-fMRI-NF*

**Figure 3:**
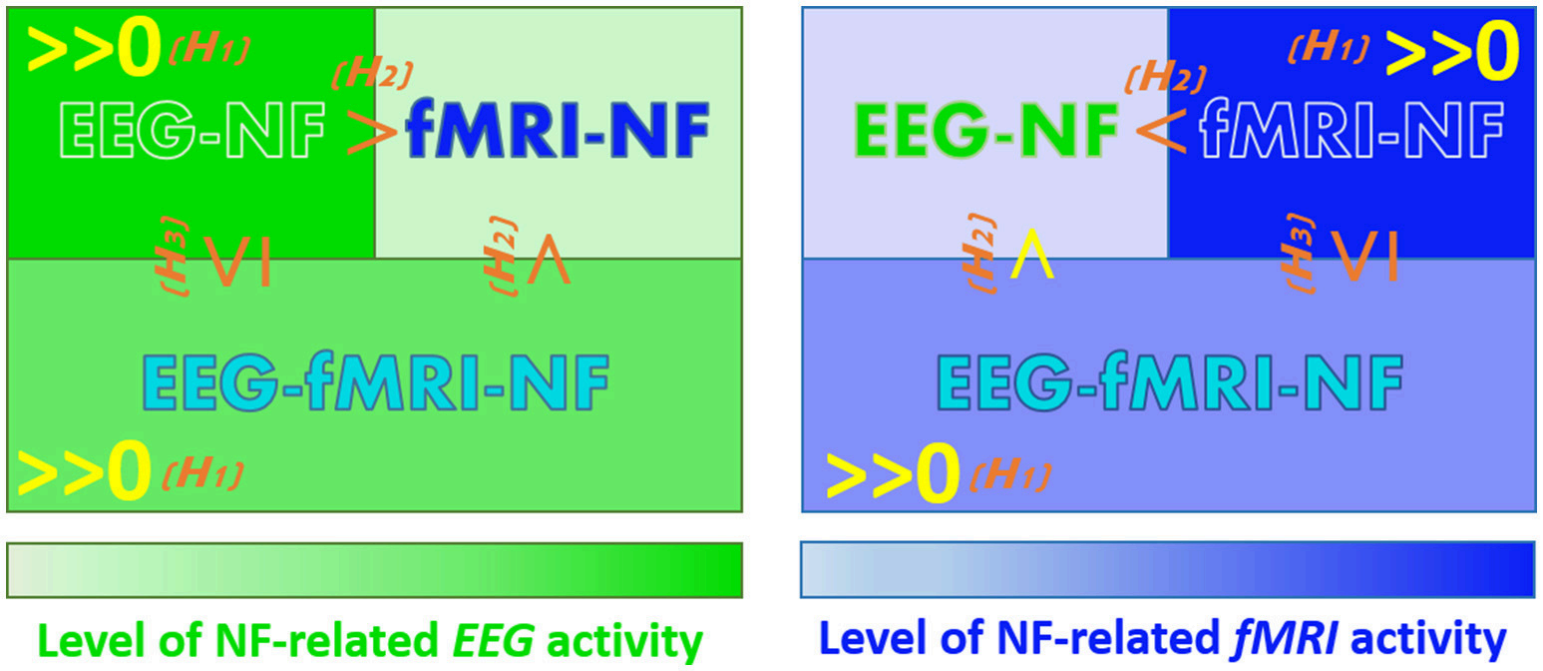
**Working hypotheses**. The hypotheses that we validated in this study are in yellow. *(H1)* Generalized NF effect. *(H2)* Direct NF effect. *(H3)* Compromise NF effect.

Figure 3 summarizes the working hypotheses.

### Offline analysis

Data from one participant was excluded because it was too affected by motion artifacts. This participant was one of the two participants for which we could not acquire the *MI_pre* and *MI_POST* data. EEG data of *MI_pre* and *MI_post* runs from one subject was accidentally lost.

#### fMRI data analysis

The fMRI data from each of the six runs (*MLOC, MI_pre, NF1, NF2, NF3, MI_post*) was pre-processed and analyzed with AutoMRI (Maumet, 2013), a proprietary software for fMRI analysis automation based on SPM8. Pre-processing included slice-time correction, spatial realignment, co-registration to the 3D T1, followed by spatial smoothing with a 8 mm Gaussian kernel. A first-level and second-level general linear model (GLM) analysis was performed. The first-level GLM included the canonical HRF for the task as well as its temporal and dispersion derivatives. For the second-level GLM analysis, the individual data were normalized to the Montreal Neurological Institute (MNI) template and grouped using a mixed effects linear model. The activations maps were corrected for multiple comparisons using Family-Wise error (FWE) correction (*p* < 0.05 with cluster size > 10 voxels).

In order to compare the level of MI-related fMRI activations between the three NF conditions, we performed a repeated measure ANOVA of the averaged offline fMRI laterality index between the three experimental conditions (A, B, and C) and paired *t*-tests between each pair of conditions. The NF blocks were averaged by considering the last six volumes (out of 10) of the blocks in order to account for the hemodynamic delay. We also performed a *post-hoc* signal analysis in order to assess the participant- and condition-specific level of activation of the actual fMRI patterns in the motor regions during NF as identified from the individual GLM analysis. For each participant, the *post-hoc* ROI was defined by running a GLM on the concatenation of *MI_pre, EEG-NF, fMRI-NF, EEG-fMRI-NF*, and *MI_post* runs (or just the NF runs for subjects who did not perform *MI_pre*) and taking a 3 × 3 × 3 box around the maximum of activation (constrained to the left motor area) of the thresholded T-map (TASK > REST, *p* < 0.05, FWE corrected, *k* > 10). For each participant and experimental condition, the registered fMRI values were high-pass filtered (100 s) to remove the linear drift, averaged in the ROI and transformed to percent signal change (PSC) using the formulae (*B*_*roi*_(*v*) − *m*)/*m* where m is the mean of all *B*_*roi*_ values across the run. Eventually, for each experimental condition the PSC were averaged across the last six volumes of each NF blocks. We then performed a repeated measure ANOVA of this *post-hoc* feature for the three experimental conditions (A, B, and C) and paired *t*-tests between each pair of conditions. In order to account for any learning effect that could have occurred throughout the consecutive runs, we also computed the repeated measure ANOVA and the paired *t*-tests on the consecutive runs. For ANOVA and paired *t*-tests, the PSC values were standardized to z-scores.

#### EEG data analysis

For offline analysis, EEG signal was pre-processed using BrainVision Analyzer II software: data was cleared from gradient and CB artifact using the artifact subtraction method (Allen et al., 2000), down-sampled to 200 Hz, filtered between 8 and 30 Hz using a Butterworth zero phase filter (48 db slope), segmented in 1 s segments, and segments affected by motion were removed. The EEG offline laterality index was then computed from this offline cleaned data in Matlab. For each of the three NF conditions (A: *EEG-NF*, B: *fMRI-NF*, C: *EEG-fMRI-NF*), we performed a repeated measure ANOVA of the averaged offline EEG laterality index between the three experimental conditions (A, B, and C) and paired *t*-tests of the averaged offline EEG laterality index. The NF blocks were averaged by considering the values between the first and the nineteenth second of the block. We also performed a *post-hoc* analysis whose purpose was to assess the participant- and condition-specific level of activation of the actual EEG patterns over the motor regions during NF as identified with a Common Spatial Pattern (CSP; Ramoser et al., 2000). For each participant, we computed the pairs of spatial filters that best maximized the difference in μ power between rest and NF blocks on the concatenation of *MI_pre, EEG-NF, fMRI-NF, EEG-fMRI-NF*, and *MI_post* (or just the NF runs for subjects who did not perform *MI_pre*) using the CSP algorithm (Ramoser et al., 2000) on 18 channels located over the motor regions (C3, C4, FC1, FC2, CP1, CP2, FC5, FC6, CP5, CP6, C1, C2, FC3, FC4, CP3, CP4, C5, C6). The first filter *f*_*rest*>*nf*_ of the pair maximizes the band power during the rest blocks while the second filter *f*_*nf*>*rest*_ of the pair maximizes the band power during the NF blocks. If the eigenvalue of *f*_*rest*>*nf*_ was greater than the inverse of the eigenvalue of *f*_*nf*>*rest*_ (Blankertz et al., 2008), the data was filtered with *f*_*rest*>*nf*_; the band power in the μ band was then computed on this filtered data using the periodogram and it was normalized with an event-related desynchronization (ERD)-like formulae $\left(\bar{REST}-bandpower\right)/\bar{REST}$ with $\bar{REST}$ being computed by averaging the power on all the baseline blocks from the run. Otherwise, the data was filtered with *f*_*nf*>*rest*_; the band power in the μ band was then computed on this filtered data using the periodogram and it was normalized with an ERD-like formulae $\left(bandpower-\;\bar{REST}\right)/\bar{REST}$ with $\bar{REST}$ being computed by averaging the power on all the baseline blocks from the run. Eventually, for each experimental condition the ERD values were averaged by considering the values between the first and the nineteenth second of each NF blocks. We then performed a repeated measure ANOVA of this *post-hoc* feature for the three experimental conditions (A, B, and C) and paired *t*-tests between each pair of conditions. In order to account for any learning effect that could have occurred throughout the consecutive runs, we also computed the repeated measure ANOVA and the paired *t*-tests on the consecutive runs. For ANOVA and paired *t*-tests, the PSC values were standardized to z-scores.

## Results

### fMRI data analysis

Whole brain analysis of the contrast TASK revealed similar networks of activations during motor execution and motor imagery with the unimodal and bimodal NF conditions.

**Figure 4:**
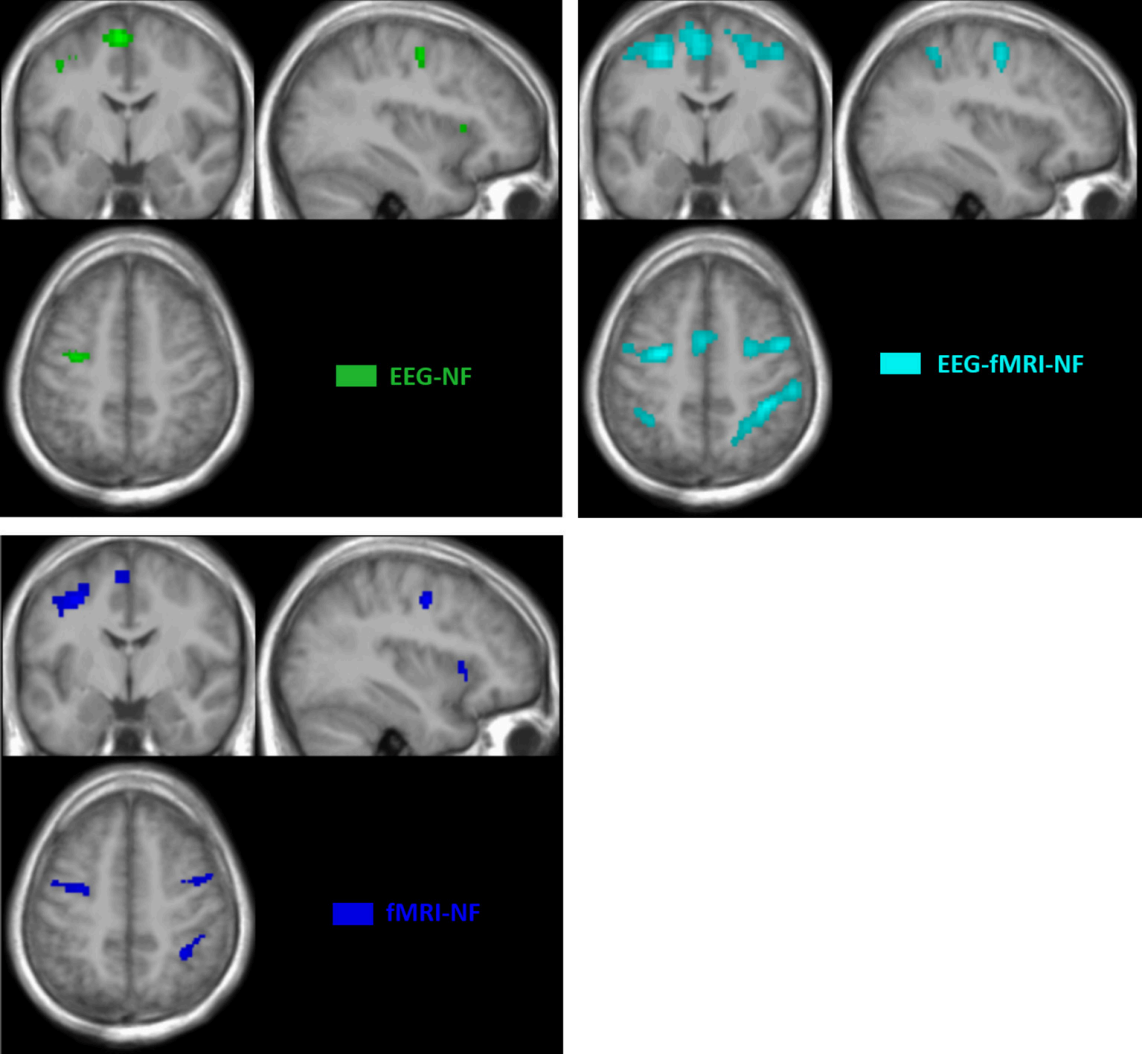
**BOLD activations maps at group level (TASK>REST; ***p*** > 0.05 FWE corrected; ***k*** > 10 voxels)**. Green, EEG-NF; Blue, fMRI-NF; Cyan, EEG-fMRI-NF. *EEG-fMRI-NF* activations are visually larger and more widespread than *EEG-NF* or *fMRI-NF* activations.

The motor execution revealed significant activations (*p* < 0.05, FWE-corrected) in the primary motor cortex (M1), in the premotor cortex and in the cerebellum. All NF conditions exhibited significant activations (*p* < 0.05, FWE corrected) in the left and right premotor cortex (PMC) and in the left and right supplementary motor area (SMA). fMRI-NF and EEG-fMRI-NF exhibited significant activations (*p* < 0.05, FWE corrected) in the right inferior frontal gyrus (pars ocularis, BA44), right inferior parietal lobule (BA40), right insula (BA47), in the right supramarginal gyrus (BA2), right superior temporal gyrus (BA42). fMRI-NF exhibited significant activations in the left insula (BA47) and in right visual cortex (BA 19). EEG-fMRI-NF exhibited significant activations in the right primary motor cortex (BA3), in the right middle temporal gyrus (BA37), left IPL (BA40). These activations are illustrated in Figure 4.

**Figure 5:**
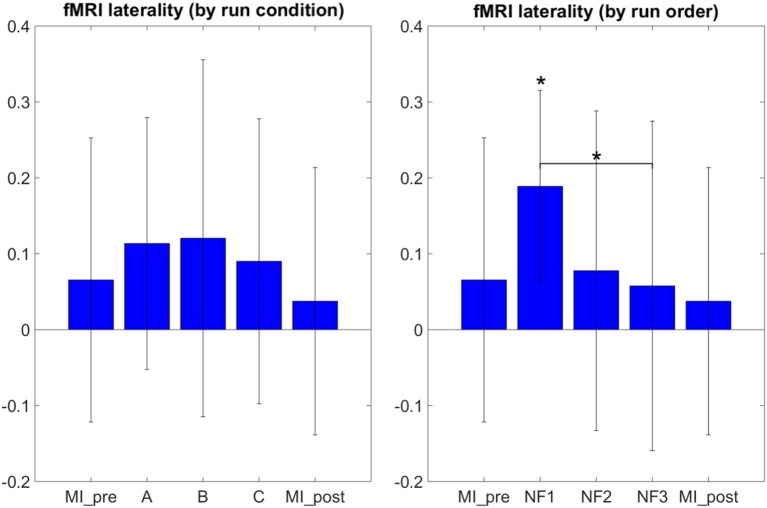
**fMRI laterality group mean with standard deviation during task in percent signal change relative to baseline**. NF conditions A (EEG-NF), B (fMRI-NF), C (EEG-fMRI-NF) were presented in different order for each subject. On the left side, the means were computed by averaging the data across subjects on each NF conditions A, B, C. On the right the means were computed by averaging the data across subjects on each NF runs by chronological order NF1, NF2, NF3. fMRI laterality was significant in the NF1 run [*t*_(8)_ = 4.1067, *p* = 0.0026]. fMRI laterality change was significantly different between NF1 and NF3 [*t*_(8)_ = 3.3351, *p* = 0.0103], which suggests that fMRI laterality tended to worsen throughout the consecutive NF runs. Stars indicate the significance level: ^*^p < 0.05.

The results in Figure 5 demonstrate that participants were able to increase their fMRI laterality between the left and right primary motor cortex during NF. The fMRI laterality change was significant in NF1 run [*t*_(8)_ = 4.4832, *p* = 0.0020]. Also, fMRI laterality change was significantly different between NF1 and NF3 [*t*_(8)_ = 3.3351, *p* = 0.0103], which suggests that fMRI laterality tended to worsen over the course of the experiment. The results in Figure 5 also illustrate that the fMRI laterality in the primary motor cortex showed high variability across subjects. Therefore the comparison between each pair of conditions did not show any significant difference. At this point, we can pinpoint that laterality features can be hard to interpret as they reflect a variety of activations patterns combining the left and right ROI (Chiew et al., 2012). For instance, in Figure 5 the higher level of activity observed during *EEG-NF* (A) as compared to *EEG-fMRI-NF* (C) is due to the fact that the group mean activity during EEG-NF was negative in the right hemisphere ROI, though it was close to zero in the left hemisphere ROI. The *post-hoc* analysis allowed to look directly at the actual activations clusters in order to assess whether there was any significant differences in the level of fMRI activity that would have hid behind the fMRI laterality measure.

**Figure 6:**
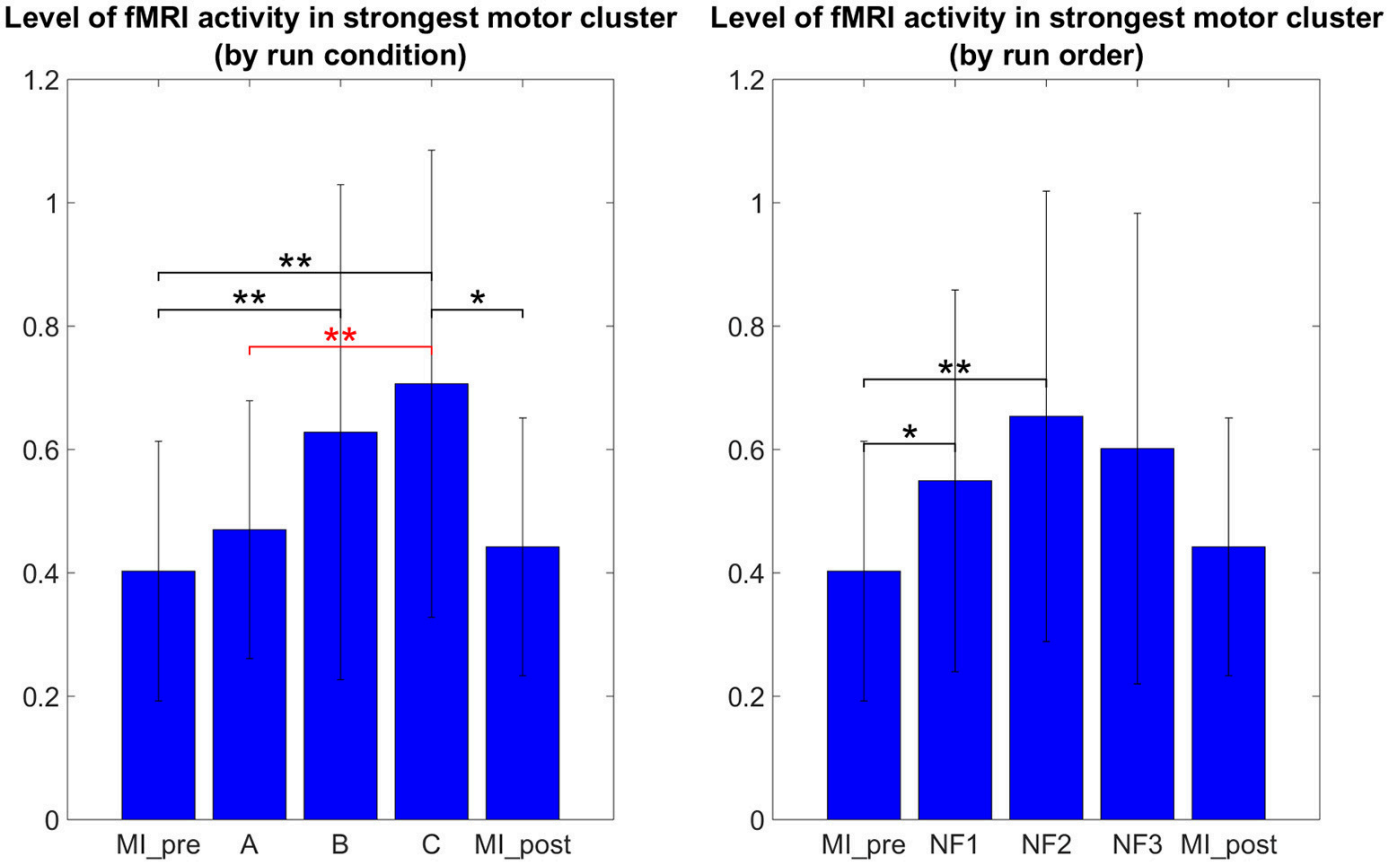
*****Post-hoc*** fMRI activations (defined as activity in strongest motor cluster after GLM) as group mean PSC during task with standard deviation**. The *post-hoc* fMRI activations were significantly higher during the *EEG-fMRI-NF* run than during the *EEG-NF* run [*t*_(8)_ = 3.8450, *p* = 0.0049]. Also *post-hoc* fMRI activations were significantly higher during motor imagery with NF than during MI without NF, which shows the added value of NF. For paired *t*-tests, PSC values were standardized to z-scores. Black significance bars were computed on eight subjects while red significance bar was computed on nine subjects. Stars indicate the significance level: ^*^p < 0.05, ^**^p < 0.01.

It is therefore not surprising that the results in Figure 6 do not show the same tendencies than the results in Figure 5 as they are direct measure of the level of activation in the actual clusters of activations instead of laterality measures. One-way repeated measure ANOVA yielded a significant effect of the NF conditions [*F*_(2, 8)_ = 5.4; *p* = 0.0162]. The results in Figure 5 show that *post-hoc* fMRI activations were significantly higher during the *EEG-fMRI-NF* condition as compared to the *EEG-NF* condition [*t*_(8)_ = 3.8450, *p* = 0.0049]. *Post-hoc* fMRI activations were significantly higher during MI with NF as compared to MI without NF, which shows the added value of NF. In particular, *post-hoc fMRI-NF* activations were significantly higher than *MI_pre* activations [*t*_(7)_ = 4.0439, *p* = 0.0049]. *EEG-fMRI-NF* activations were significantly higher than *MI_pre* activations [*t*_(7)_ = 4.2320, *p* = 0.0039] and significantly higher than *MI_post* activations [*t*_(7)_ = 2.8855, *p* = 0.0235]. *NF1* activations were significantly higher than *MI_pre* activations [*t*_(7)_ = 3.4530, *p* = 0.0106]. *NF2* activations were significantly higher than *MI_pre* activations [*t*_(7)_ = 3.8277, *p* = 0.0.0065]. Results are summarized in Figure 9.

### EEG data analysis

**Figure 7:**
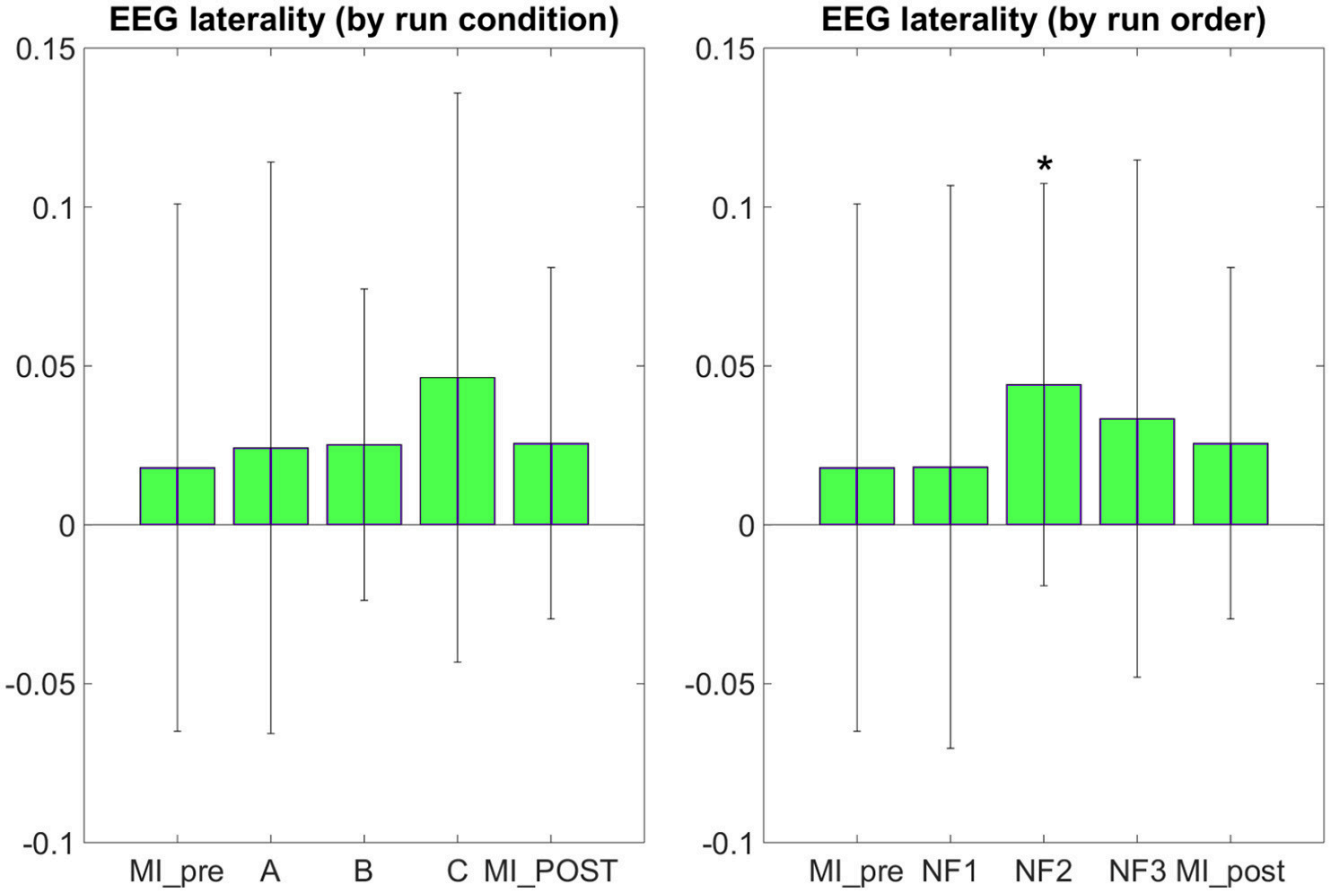
**EEG laterality group mean with standard deviation during task in percent signal change relative to baseline**. NF conditions A (EEG-NF), B (fMRI-NF), C (EEG-fMRI-NF) were presented in different order for each subject. On the left, the means are computed by averaging the data across subjects on each NF conditions A, B, C. On the right the means are computed by averaging the data across subjects on each NF runs by chronological order NF1, NF2, NF3. EEG laterality was significant in the second NF run [*t*_(8)_ = 2.3389, *p* = 0.0441]. Though not significant, we observe that the EEG laterality tended to improve over the course of the experiment. Stars indicate the significance level: ^*^p < 0.05.

**Figure 8:**
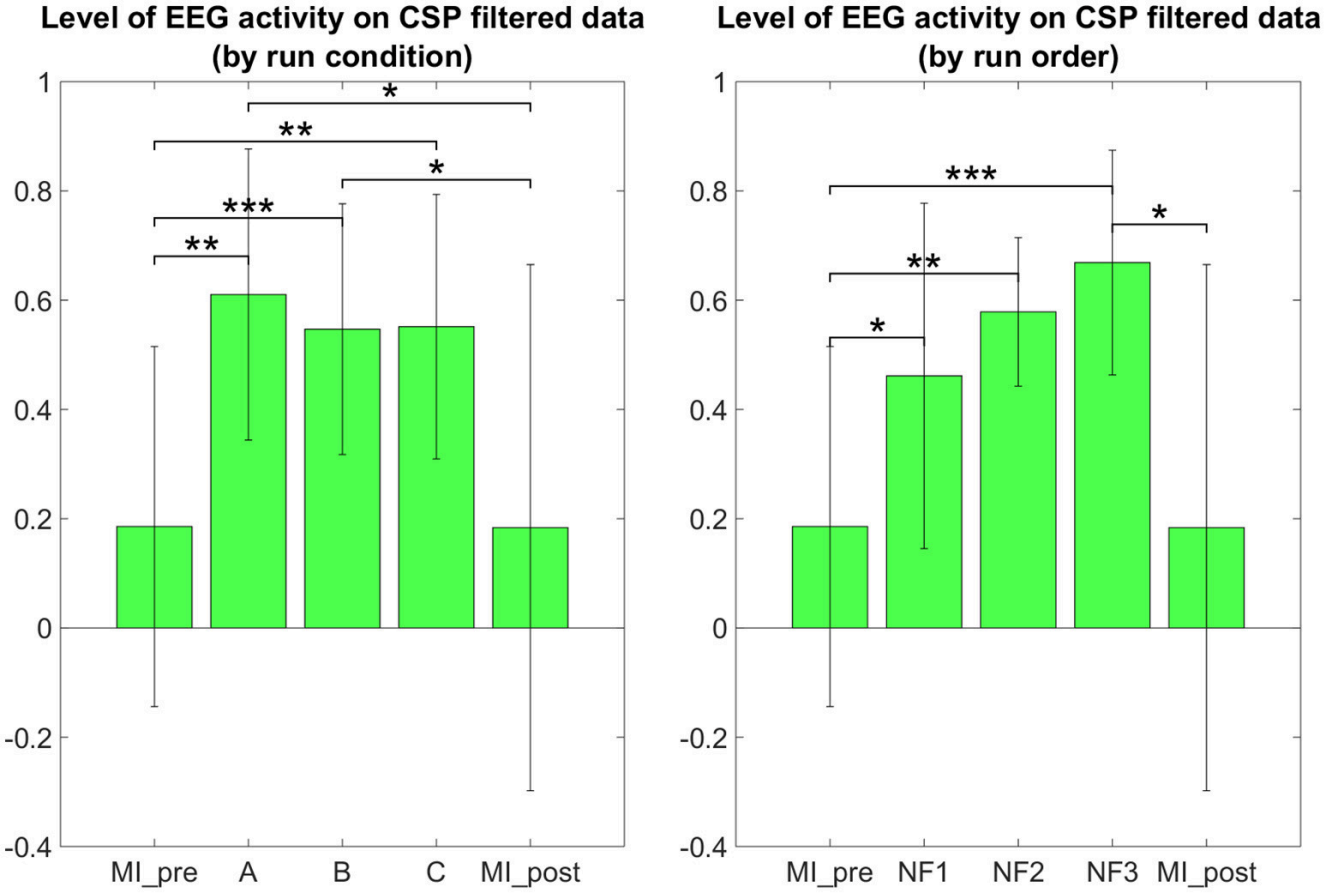
*****Post-hoc*** EEG activations group mean ERD in the μ band after CSP filtering**. *Post-hoc* EEG activations were significantly higher during motor imagery with NF than during MI without NF, which shows the added value of NF. There was no significant differences between the three NF conditions (A, B, C) nor between the three NF runs (NF1, NF2, NF3). For paired *t*-tests, ERD values were standardized to z-scores. Black significance bars were computed on seven subjects. Stars indicate the significance level: ^*^p < 0.05, ^**^p < 0.01, ^***^p < 0.001.

**Figure 9:**
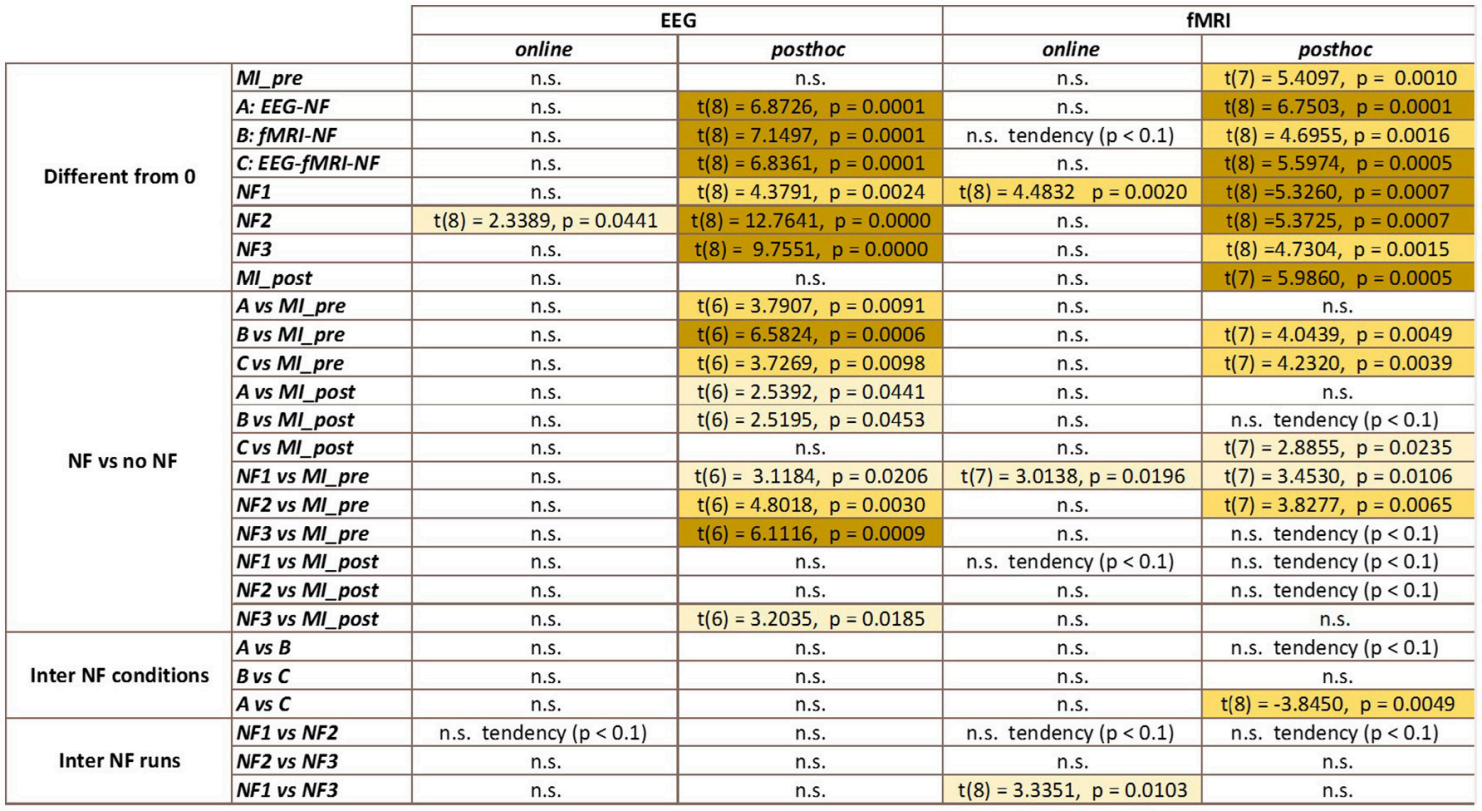
**Summary of the statistical analysis results (***t***-tests and paired ***t***-tests)**. Color indicates the level of significance of the tests.

The results in Figure 7 demonstrate that participants were able to increase their EEG laterality between C1 and C2 in the μ band during NF. The EEG laterality change was significant in the second NF run [*t*_(8)_ = 2.3389, *p* = 0.0441]. These results also suggest, however without significance, that EEG laterality tended to improve over the course of the experiment. As for the fMRI laterality feature, the EEG laterality between C1 and C2 in the μ band showed high variability across subjects. Therefore, the comparison between each pair of conditions did not show any significant difference. The *post-hoc* analysis aimed at looking directly at the actual EEG patterns of activity in order to assess whether there was any significant differences that would have hid behind the EEG laterality measure. However, as illustrated in Figure 8, *post-hoc* EEG activations did not show any significant differences between the NF conditions either. *Post-hoc* EEG activations were significantly higher during MI with NF as compared to MI without NF, which shows the added value of NF. In particular, *post-hoc EEG-NF* activations were significantly higher than *MI_pre* activations [*t*_(6)_ = 3.7907, *p* = 0.0091] and significantly higher than *MI_post* activations [*t*_(6)_ = 2.5392, *p* = 0.0441]. *Post-hoc fMRI-NF* activations were significantly higher than *MI_pre* activations [*t*_(6)_ = 6.5824, *p* = 0.0006] and significantly higher than *MI_post* activations [*t*_(6)_ = 2.5195, *p* = 0.0453]. *Post-hoc EEG-fMRI-NF* activations were significantly higher than *MI_pre* activations [*t*_(6)_ = 3.7269, *p* = 0.0098]. *NF1* activations were significantly higher than *MI_pre* activations [*t*_(6)_ = 3.1184, *p* = 0.0206]. *NF2* activations were significantly higher than *MI_pre* activations [*t*_(6)_ = 4.8018, *p* = 0.0030]. *NF3* activations were significantly higher than *MI_pre* activations [*t*_(6)_ = 6.1116, *p* = 0.0009] and significantly higher than *MI_post* activations [*t*_(6)_ = 3.2035, *p* = 0.0185]. Results are summarized in Figure 9.

### Questionnaire

In the questionnaire participants were asked specific questions about the *EEG-fMRI-NF* run. Seven participants out of 10 reported that they did not feel like they had to perform two regulation tasks. Six participants found that fMRI was easier to control than EEG; three found that EEG was easier; one found no difference. Eight participants out of ten reported to have paid the same attention to both dimensions during the *EEG-fMRI-NF* condition, the two others reported they looked more are the dimension that was harder for them to control (in one case EEG, in the other fMRI). Five participants out of 10 reported that fMRI update rate was slow.

## Discussion

For the first time, we compared the effects of unimodal *EEG-NF* and *fMRI-NF* with bimodal *EEG-fMRI-NF* in order to assess the potential added value of bimodal NF over unimodal NF. We tested our hypotheses (cf. Section Working Hypotheses) by looking at the level of MI-related EEG and fMRI activations during each NF conditions. Motor activations as revealed by *post-hoc* fMRI analysis were significantly higher during *EEG-fMRI-NF* than during *EEG-NF* (see Figure 6). This results partly validated our “direct NF effect” hypothesis and could mean that *EEG-fMRI-NF* specifically triggered more fMRI activations than *EEG-NF* because feedback from fMRI was provided. But it could also mean that bimodal *EEG-fMRI-NF* was more engaging than unimodal *EEG-NF* because subject had to control the feedback in the vertical and horizontal dimension. To disentangle whether *EEG-fMRI-NF* is more specific or simply more engaging than *EEG-NF*, one could use a one-dimensional *EEG-fMRI-NF* feedback that would mix both EEG and fMRI feature in a single gauge and compare it directly to *EEG-NF*. Alternatively, to rule out the engaging factor, one could also compare *EEG-fMRI-NF* with EEG-shamfMRI-NF in which sham fMRI-NF would be provided together with real EEG-NF. *Post-hoc* EEG activations did not show any significant differences between the different NF conditions. This can be due to the fact that EEG is noisier than fMRI, especially in the MR environment, but it is also possible that it was hard for participants to maintain the μ desynchronization throughout the 20 s of the NF blocks. The 20 s block design was chosen mainly in consideration of the fMRI modality in order to account for the hemodynamic delay. MI-based EEG-NF/-BCI tasks are usually much shorter, around 4 s length (Pfurtscheller and Neuper, 2001). The electrophysiology of continuous MI is still not fully understood. Though continuous MI is thought to induce a succession of ERDs it can be hard to observe a continuous desynchronization throughout the duration of the continuous MI (Jeon et al., 2011; Rimbert et al., 2015) This highlights the difficulty of designing the task specifically for bimodal *EEG-fMRI-NF* given the different spatio-temporal dynamics of EEG and fMRI. Interestingly, the specific effect of NF in the three NF conditions can be confirmed by the significant difference in the level of *post-hoc* fMRI and EEG motor activations between the NF runs and the *MI_pre* and *MI_post* runs which were done without NF (see Figures 6, 8). Despite the somehow limited number of subject in our study, these results support our “generalized NF effect” hypothesis. Further work with more subjects should be conducted to even enforce this outcome. In the seminal work on EEG-fMRI-NF (Zotev et al., 2014), the authors studied a protocol of positive emotion induction with feedback from frontal EEG asymmetry in the beta band and from left amygdala BOLD. As in this related work, we found similar value ranges of the EEG and fMRI features and similar variability. We were however not able to observe significant differences between the three NF conditions by directly looking at the EEG and fMRI laterality features (see Figures 5, 7). Lateralization of activity in motor regions is known to be an indicator of good motor imagery (Marchesotti et al., 2016). Also in stroke rehabilitation, best results are usually obtained when the recovery happens in the ipsi-lesional hemisphere rather than in the contra-lesional one and NF based on laterality indices could allow to promote this kind of recovery (Chiew et al., 2012; Rehme et al., 2012). However, laterality features are hard to interpret and may have been too hard to regulate significantly in a single session for participants who were not trained to MI before. Regarding the EEG laterality index and given the spatial proximity of the chosen electrode locations C1 and C2, one could wonder if they could be influenced by the same sources. Such sources would be situated in cortices close to the inter-hemispherical midline such as feet sensorimotor area. However, the activity of the hand sensorimotor area is quite far from the midline, so the activity measured by a contralateral electrode will be far stronger than that of an ipsilateral electrode. Given that the neurofeedback was based on a laterality index, there is no chance that activating common sources such as with feet imagination would allow to control the neurofeedback. However, we do admit that C3 and C4 are more common locations for hand movements and might lead to better results. One could also consider computing a CSP filter on calibration data which would allow to define the spatial filtering for the EEG feature at the individual level. Regarding the fMRI laterality index, the right motor ROI was defined approximately by mirroring the left motor ROI. This was done mainly in order not to add more time to the already long experimental protocol. Given the size of the ROI (18 × 18 × 12 mm^3^), there is high chance that the mirror ROI would lie in the right primary motor cortex. However, we admit that it would be better to use a functional localizer to define the right motor ROI.

Regarding the “compromise NF effect,” our results did not allow us to get any preliminary insight into our speculations. More experiments with longer NF training and more subjects are needed to confirm the rest of the “direct NF effect” and the “compromise NF effect” assumptions. We can note that in our study, the signal-to-noise ratio (SNR) was the same in unimodal and bimodal NF conditions as EEG and fMRI were simultaneously acquired throughout the whole experiments to assess the cross-modality effects. However, when doing unimodal EEG-NF or fMRI-NF without simultaneous EEG and fMRI recordings, SNR should be better than the one of bimodal EEG-fMRI-NF. This could reinforce the “compromise NF effect.” Artifacts occurring during simultaneous EEG-fMRI are a major limitation of EEG-fMRI-NF (Zotev et al., 2014). The BCG artifact and motion artifacts from the subject or the environment (vibrations from helium pump and ventilation) are particularly hard to correct. The development of new methods for correcting these artifacts is an ongoing topic of research, but few options are available for online correction (Allen et al., 1998, 2000; Krishnaswamy et al., 2016; Mayeli et al., 2016; Wu et al., 2016; van der Meer et al., 2016). Interestingly, a recent approach consists in using the EEG not only as a brain imaging modality but also as a motion sensor to correct for motion artifact (Jorge et al., 2015; Wong et al., 2016). Another important aspect of the EEG analysis is the choice of the reference. In this work we used the standard reference FCz as it was proven to be efficient for motor imagery (Choi et al., 2006). But regarding the fact that the potential of FCz is non-zero, it would be interesting in the future to consider using another reference such as the common average reference (CAR) or reference electrode standardization technique (REST; Yao, 2001).

Though the NF features change between the consecutive NF runs and between each pair of NF conditions was not significant, the EEG and fMRI laterality features had asymmetric tendency (see Figures 5, 7). Throughout the consecutive NF runs, EEG laterality tended to improve while fMRI laterality tended to worsen. Besides, participants reported on average that the fMRI dimension was easier to control than the EEG dimension, so it is possible that they have put more effort (however not necessarily more attention as they reported) on controlling the EEG dimension. This could explain the learning tendency observed on the EEG laterality feature at the price of a decrease on the fMRI laterality feature. Putting these observations together suggests that during bimodal NF, one feature could be more regulated than the other, possibly the one that is harder to control. We should note however that our study was conducted at a single-session level and that the asymmetric change of the features that we observed could actually be part of a learning scenario in which subjects would by example first learn to regulate one feature, then the other one and eventually manage to regulate both simultaneously. Interestingly, this decrease of performance on NF features was also observed in related works (Zotev et al., 2014) though both on EEG and fMRI features. This decrease of performance can also be explained as being part of the U-shaped learning curve (Carlucci and Case, 2013; Gaume et al., 2016): by trying new regulation strategies, the cognitive load of the subject suddenly increases and results in a temporary loss of performance. However, it is not yet known how this applies to bimodal NF. Our results thus open interesting questions on how subjects learn to regulate a bimodal NF and on how to define the EEG and fMRI features so that they are complementary enough. The assessment of this complementarity can be based on studies and methods investigating the coupling between BOLD and EEG signal (Formaggio et al., 2010; Yuan et al., 2010; Dong et al., 2014; Murta et al., 2015, 2016; Yin et al., 2016) which generally report that BOLD is negatively correlated with low-frequency EEG bands (α, β) and positively correlated with high-frequency EEG bands (γ). Besides these questions on the learning mechanisms and the inner definition of the features, our observations also raise the issue of whether the two NF signals should be made discriminable or not by the feedback metaphor. Indeed, if the subject was not able to discriminate between both signals, he/she might be less likely to control one signal more than the other.

Feedback design is an important aspect of a neurofeedback protocol and the optimal form of feedback for unimodal NF is still an ongoing topic of research (Cohen et al., 2016). Though the traditional thermometer metaphor (Sitaram et al., 2007) can appear boring for subjects, it has the advantage of being easy to understand. In their pioneering work, Zotev et al. (2014) have naturally extended the thermometer feedback to the bimodal NF case. We introduced a novel metaphor for EEG-fMRI-NF that integrates both signal into one single feedback in order for the subject to more easily perceive the bimodal NF task as one single regulation task. Though we did not compare our integrated metaphor with a non-integrated one, most of our participants reported that it felt like they had one task to do during bimodal NF. Having two separate feedbacks to control and thus two separate targets to achieve could increase the cognitive load, which is an important aspect of the NF process (Gaume et al., 2016). Integrating both NF signals in one single feedback can be a way to relieve the cognitive load of the subject. One of the difficulty in combining both NF signals in a single feedback is that EEG and fMRI do not have the same sampling rate. In the present study, the fact that the update rates of the EEG and fMRI dimensions were different might have been disturbing for the participants. Indeed, five participants found that fMRI update rate was slow (16 times slower than EEG). Bringing the EEG and fMRI update rates closer is therefore advisable for future experiments. However, for fMRI, the update rate is constrained by the TR, which cannot be brought much below 1 s. One way to prevent the subject from being disturbed by the different update rates of the two modalities could be to mix the two NF signals in a feedback that would not allow the subject to discriminate between the two signals, like a one-dimensional feedback. Besides the representative advantage of using an integrated feedback metaphor, we believe that it makes it possible to define a truly integrated NF target that would reward brain patterns defined from both modalities. There is different level of “integration” of EEG and fMRI data. In our study, we integrated the two neurofeedback signals in one feedback metaphor in order to provide a bimodal neurofeedback. A more advanced way to provide an integrated bimodal feedback could be to use EEG-fMRI integration methods (Jorge et al., 2013; Sulzer et al., 2013), such as fMRI-informed EEG analysis, EEG-informed fMRI analysis, or EEG-fMRI fusion. However, despite the wide range of existing methods, these methods are mostly designed for offline use and there is no prospect yet of doing this integration online. In the framework of EEG-fMRI-NF, one could benefit from using these methods offline to study the effects of neurofeedback, guide the choice of the NF features, learn priors for a reconstruction model, learn a predictive (Meir-Hasson et al., 2013) or a coupling model.

It is important to stress that in our experiment unimodal and bimodal NF targets were different. The EEG-fMRI-NF target was probably “harder” to reach than the EEG-NF or the fMRI-NF target, as subjects needed to regulate EEG and fMRI simultaneously to reach the target. Thus, by directly integrating the EEG and fMRI NF signals without any fancy fusion technique, brain patterns defined this way from both modalities should already be more specific than those defined from one modality alone. Future experiments involving more subjects and other cognitive tasks will allow to characterize more precisely how EEG and fMRI are modulated in different unimodal and bimodal NF conditions. Eventually, the use of offline EEG-fMRI integration techniques should help understand how to define bimodal EEG-fMRI-NF protocol that make the most of both modalities for therapeutic applications such as stroke, depression, and other psychiatric and neurological disorders.

## Conclusion

We have found that MI-related hemodynamic and electrophysiological activity are modulated both during unimodal EEG-NF and fMRI-NF and during bimodal EEG-fMRI-NF. Notably, we found that MI-related hemodynamic activity was higher during EEG-fMRI-NF than during EEG-NF, unlike fMRI-NF. This result suggests that EEG-fMRI-NF could be more specific or more engaging than EEG-NF alone. We have also observed that during EEG-fMRI-NF one modality could be more regulated than the other, suggesting the existence of self-regulating processes that would be proper to bimodal NF training. Taken together, our results pave the way to novel combinations of EEG and fMRI modalities for more effective neurofeedback approaches.

## Ethics statement

This study was carried out in accordance with the recommendations of Comité de Protection des Personnes “Ouest V” Rennes with written informed consent from all subjects. All subjects gave written informed consent in accordance with the Declaration of Helsinki. The protocol was approved by the Comité de Protection des Personnes “Ouest V” Rennes.

## Author contributions

LP, AL, CB, MM, EB, FL, and MC designed the study and the unimodal and bimodal neurofeedback protocols. MM developed the real-time EEG-fMRI neurofeedback platform. LP and MM conducted the experiments. LP performed the data analysis. LP wrote the majority of the manuscript. All the authors read, revised, and approved the final manuscript.

## Funding

This work has received a French government support granted to the CominLabs excellence laboratory and managed by the National Research Agency in the “Investing for the Future” program under reference ANR-10-LABX-07-01. It was also financed by Brittany region under HEMISFER project, and the National Research Agency with the REBEL project and grant ANR-15-CE23-0013-01. MRI data acquisition was supported by the Neurinfo MRI research facility from the University of Rennes I. Neurinfo is granted by the European Union (FEDER), the French State, the Brittany Council, Rennes.

### Conflict of interest statement

The authors declare that the research was conducted in the absence of any commercial or financial relationships that could be construed as a potential conflict of interest.
